# *NPM1* Mutation Analysis in Acute Myeloid Leukemia: Comparison of Three Techniques - Sanger Sequencing, Pyrosequencing, and Real-Time Polymerase Chain Reaction

**DOI:** 10.4274/tjh.2017.0095

**Published:** 2018-03-06

**Authors:** Dushyant Kumar, Anurag Mehta, Manoj Kumar Panigrahi, Sukanta Nath, Kandarpa Kumar Saikia

**Affiliations:** 1 Gauhati University Faculty of Medicine, Department of Bioengineering and Technology, Guwahati, India; 2 Rajiv Gandhi Cancer Institute and Research Centre, New Delhi, India

**Keywords:** NPM1, Pyrosequencing, Acute myeloid leukemia, Mutation analysis

## Abstract

**Objective:**

Nucleophosmin-1 (NPM1) mutations have prognostic importance in acute myeloid leukemia (AML) patients with intermediate-risk karyotype at diagnosis. Approximately 30% of newly diagnosed cytogenetically normal AML (CN-AML) patients harbor the NPM1 mutation in India. In this study we compared the efficiency of three molecular techniques in detecting NPM1 mutation in peripheral blood and bone marrow samples.

**Materials and Methods:**

In a single-center cohort we analyzed 165 CN-AML bone marrow/peripheral blood samples for NPM1 mutation analysis. About 30% of the CN-AML samples revealed NPM1 mutations. For the detection, three methods were compared: Sanger sequencing, pyrosequencing, and real-time polymerase chain reaction (PCR).

**Results:**

NPM1 exon 12 mutations were observed in 52 (31.51%) of all CN-AML cases. The sensitivity of Sanger sequencing, pyrosequencing, and real-time PCR was 80%, 90%, and 95%, whereas specificity was 95%, 100%, and 100%, respectively. The minimum limit of mutation detection was 20%-30% for Sanger sequencing, 1%-5% for pyrosequencing, and 0.1%-1% for real-time PCR.

**Conclusion:**

The sequencing method, which is the reference method, has the lowest sensitivity and is sometimes difficult to interpret. Real-time PCR is a highly sensitive method for mutation detection but is limited for specific mutation types. In our study, pyrosequencing emerged as the most suitable technique for the detection of NPM1 mutations on the basis of its easy interpretation and less time-consuming processes than Sanger sequencing.

## Abstract (tr)

**Amaç:**

Nükleofosmin-1 (NPM1) mutasyonları tanı anında orta risk akut miyeloid lösemi (AML) hastalarında prognostik öneme sahiptir. Hindistan’da, yeni teşhis normal sitogenetiğe sahip AML (CN-AML) hastalarının yaklaşık %30’u NPM1 pozitiftir. Bu çalışmada periferik kan ve kemik iliği örneklerinde NPM1 mutasyonu saptamada kullanılan üç moleküler tekniğin etkinliğini karşılaştırdık.

**Gereç ve Yöntemler:**

Tek merkezli bu kohortta, 165 CN-AML kemik iliği/periferik kan örneklerinde NPM1 mutasyon analizi yapıldı. CN-AML örneklerinin yaklaşık %30’unda NPM1 mutasyonu saptandı. Mutasyonun taranmasında üç yöntem karşılaştırıldı: Sanger dizileme, pirodizileme, gerçek-zamanlı polimeraz zincir reaksiyonu (PCR).

**Bulgular:**

Tüm CN-AML olgularının 52’sinde (%31,51) NPM1 exon12 mutasyonları gözlendi. Sanger dizileme, pirodizileme ve gerçek zamanlı PCR’nin duyarlılıkları sırasıyla %80, %90 ve %95 iken, özgünlükleri %95, %100 ve %100’dü. Mutasyonun saptanmasında minimum limit Sanger dizileme yöntemi için %20-%30, pirodizilemede %1-5, ve gerçek-zamanlı PCR için %0,1-%1 idi.

**Sonuç:**

Referans yöntemi olan dizileme yöntemi, en düşük duyarlılığa sahiptir ve bazen yorumlaması güçtür. Gerçek-zamanlı PCR mutasyon saptamada yüksek duyarlılığa sahip bir yöntemdir fakat özel mutasyon tipleri için sınırlıdır. Çalışmamızda, pirodizileme yönteminin kolay yorumlanması ve Sanger dizileme yönteminden daha az zaman harcanan işlem olması esasına dayanarak NPM1 mutasyonun saptanmasında en uygun teknik olduğu sonucuna varılmıştır.

## Introduction

An increasing number of genetic abnormalities are revealed in acute myeloid leukemia (AML). Among these genetic alterations, potential prognostic genetic markers are the nucleophosmin 1 (*NPM1*) gene,* FLT3 *gene, and *CEBPA *gene [1]. Mutations in the* NPM1 *and *FLT3 *genes represent the most important diagnostic and prognostic indicators in patients with cytogenetically normal AML (CN-AML). *NPM1* is a phosphoprotein that continuously shuttles between the cytoplasm and nucleus. Several functions for this protein have been described, including the binding of* p53*, the initiation of centrosome duplication, and ribosomal protein assembly and transport [2].* NPM1 *mutations found in exon 12 code for the COOH terminal region. Frameshift mutations in the *NPM1 *gene result in an elongated protein that contains an additional nuclear export signal and leads to an abnormal cytoplasmic localization of the protein [3,4]. These mutations are involved in leukemogenesis and are detected in about 35%-60% of AML cases [5]. Six types of* NPM1 *mutation variants have been identified: *NPM1 *mutation A (*c.860_863dupTCTG*), mutation B (*c.862_863insCATG*), mutation D (*c.863_864insCCTG*), mutation I (*c.863_864insTAAG*), mutation J (*c.863_864insCTTG*), and mutation K (*c.863_864insTATG*). Mutation A (TCTG insertion) is the most commonly occurring variant, found in about 80% of all* NPM1*-mutated AML cases (Table 1) [3,5]. The effect of mutant* NPM1 *has been studied using gene expression profiling and studies revealed a distinctive signature of these mutations [6]. Many studies reported the prognostic significance of* NPM1 *mutation status in AML [7,8,9,10,11]. There are highly specific and sensitive molecular assays available for detecting* NPM1 *mutations, like Sanger sequencing, high-resolution melting curve analysis, real-time polymerase chain reaction (PCR), and pyrosequencing (Pyr). In this study, we evaluated the utility of Pyr in the detection of* NPM1 *mutation detection and also compared it with Sanger sequencing and real-time PCR in terms of assay sensitivity, specificity, limit of mutation detection, turnaround time, and assay cost [12,13].

## Materials and Methods

A total of 165 CN-AML bone marrow aspiration or peripheral blood samples taken at the time of first diagnosis were included in this study from February 2014 to September 2016. Out of these 165 patients, 79 (47.87%) were male and 86 (52.12%) were female. Twenty cases (12.12%) were pediatric cases.

### DNA Extraction

Genomic DNA was extracted from the received samples using the QIAGEN DNeasy Kit (QIAGEN, Hilden, Germany) as per the manufacturer’s instructions.

### *NPM1* Mutation Detection by Pyr Analysis

In the Pyr method for DNA sequence analysis, inorganic phosphate released in the course of nucleotide incorporation serves as the initial substrate in a sequence of four successive enzymatic reactions. This results in the emission of light, which functions as a signal that is proportional to the number of nucleotides incorporated.

For *NPM1* mutation analysis TTAACTCTCTGGTGGTAGAATG was used as a forward primer, biotin-ACATTTATCAAACACGGTAGG as a reverse primer, and TTTTCCAGGCTATTCAAGAT as the sequencing primer (Sigma-Aldrich, New Delhi, India). DNA (50 ng) was amplified using 400 nmol of forward and reverse primers in 25 µL of reaction mix with PyroMark master mix (QIAGEN). PCR conditions were as follows: initial denaturing at 95 °C for 15 min; 42 cycles of 95 °C for 20 s, 53 °C for 30 s, and 60 °C for 20 s; and final extension at 72 °C for 5 min. PCR products were electrophoresed on agarose gel to confirm successful amplification. The PCR products (10 µL) were then sequenced with the Pyr PyroMark Q24 system (QIAGEN).

### *NPM1* Mutation Detection by Pyr Analysis Using Ipsogen *NPM1* MutaScreen Kit

The Ipsogen* NPM1 *MutaScreen Kit (QIAGEN) combines two techniques to screen for the presence of mutations in the target gene. The real-time quantitative PCR (qPCR) double-dye oligonucleotide hydrolysis principle uses specific primers and an internal double-dye probe with a reporter and a quencher (FAM-TAMRA) for the amplification reactions. In addition, a 3’-end modified phosphate oligonucleotide is used that perfectly matches the wild-type *NPM1* gene and does not allow polymerization. The Ipsogen* NPM1 *MutaScreen Kit detects total* NPM1 *(wild-type + mutated) and mutated* NPM1 *and separately identifies* NPM1 *Mut A, Mut B, and Mut D in genomic DNA. A sample of DNA of 25 ng was used in a final reaction volume of 25 µL. The PCR profile for Rotor-Gene Q (QIAGEN) was 50 °C for 2 min, 95 °C for 10 min, and then 40 cycles of 95 °C for 15 s and 60 °C for 90 s with acquisition performed at 60 °C. Analysis was performed as per the kit’s instructions.

### *NPM1* Mutation Analysis by Sanger Sequencing

Analysis of *NPM1* exon 12 mutations was done as described by Falini et al. [4]. A sample of DNA of 50 ng was amplified using an Applied Biosystems Veriti thermal cycler (Foster City, CA, USA) and purified PCR product was used for BigDye termination bidirectional sequencing. Results were analyzed using BioEdit sequence analysis software.

## Results

*NPM1 *exon 12 mutation was observed in 52 (31.51%) of all CN-AML cases. As expected, the percentage of the DNA samples in which mutations were detected varied and depended upon the method of detection used. *NPM1 *mutation analysis by Pyr had the highest likelihood of identifying a mutation in the* NPM1 *gene, followed by the* NPM1* MutaScreen kit and direct sequencing (Table 2). However, on the basis of our evaluation criteria (Table 1), the most sensitive tool was the Ipsogen MutaScreen kit (95%), followed by Pyr (90%) and Sanger sequencing (80%). In terms of specificity, all three methods matched equally.

## Discussion

**Figure 1:**
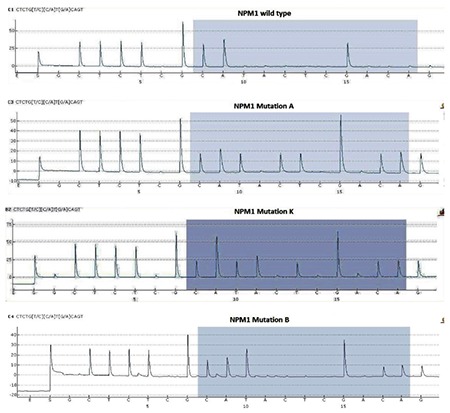
*NPM1* mutation detection by pyrosequencing detection by pyrosequencing.

It has been found that 99% of all* NPM1 *mutations detected by Pyr have 4-base insertions at position 860 while the rest of the* NPM1 *mutations detected by Pyr were found as insertion at 862 and deletion at 863 and 861 [14]. We have examined the ability of three different methods to detect mutations in *NPM1 *gene exon 12 in 165 CN-AML samples. Bone marrow or peripheral blood samples with a minimum of 15% blasts were examined in this study. *NPM1 *mutations were found in 52 samples (31.51%), while 113 (68.48%) samples were found to be wild-type. Twenty-eight (53.84%) of the* NPM1*-positive patients were male while 24 (46.15%) were female. Seven (13.46%) of the *NPM1*-positive samples were from pediatric patients while 45 (86.53%) were from adults. Mutation type A was the most frequent mutation (~80%), followed by types B (12%) and D (6%). We also found one case of mutation type K (*c.863_864insTATG*) by Pyr (Figure 1). The sequencing method is considered the gold-standard technique for detection of somatic as well as generic mutations. Jancik et al. [15] compared the specificity, sensitivity, cost, and working time of five techniques including Pyr, Sanger sequencing, and real-time PCR for *KRAS* mutations. Ogino et al. [16] stated that the Pyr assay to detect somatic mutations from formalin-fixed paraffin embedded tissue is more sensitive than Sanger sequencing. Tsiatis et al. [17] compared Pyr, Sanger sequencing, and melting curve methods for the detection of somatic mutations like *KRAS*, *NRAS*, and *BRAF* and demonstrated that Sanger sequencing specificity is generally high compared with other methods, but sensitivity has been reported to differ. Real-time PCR is the most sensitive method for detecting minimal residual disease [18], but it is limited to specific detection of mutations A, B, and D. In the case of limited mutation, we can synthesize primers and probes for other mutations as well, but it will add extra cost per reaction (Table 3).

**Figure 2:**
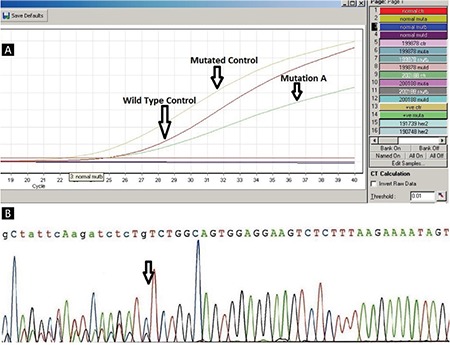
A) *NPM1* mutation detection by real-time polymerase chain reaction using Ipsogen MutaScreen Kit. B) Sanger sequencing.

Pyr is easily capable of detecting PCR fragments that are 25-50 bp in length while longer fragments may pose a problem [15,16]. In the case of* NPM1*,in which 99% of mutations occur at position 956 in exon 12 [14], with Pyr we were able to detect all types of mutations (Figure 1) with lower cost than real-time PCR and less time than Sanger sequencing (Figure 2). Recently next-generation sequencing (NGS) has become popular for detection of mutations in 50 genes to 100 genes simultaneously. NGS is the method to detect mutations down to the mutational burden of 1.25%. However, even though NGS is an accurate method, it is still costly and time-consuming compared with Pyr.

## Conclusion

In our study Pyr emerged as the most suitable technique for the detection of* NPM1 *mutations on the basis of its easy interpretation and less time-consuming processes than Sanger sequencing. However, the limit of mutation detection by real-time PCR is 0.1%-1%, the lowest of all three techniques, so real-time PCR is the best technique to determine minimal residual disease compared to Pyr, which has a limit of detection of 1%-5%. The Pyr assay can be considered as a better technique for *NPM1* mutation detection.

## Figures and Tables

**Table 1:**
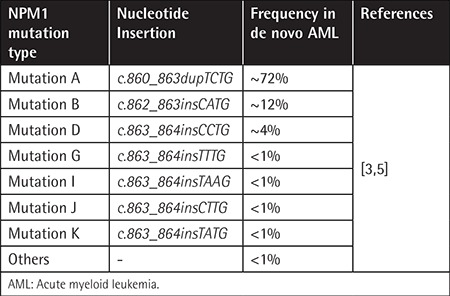
*NPM1* mutation frequencies in de novo acute myeloid leukemia.

**Table 2:**
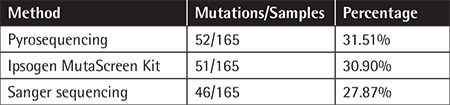
Number and percentage of mutations detected by three different methods.

**Table 3:**
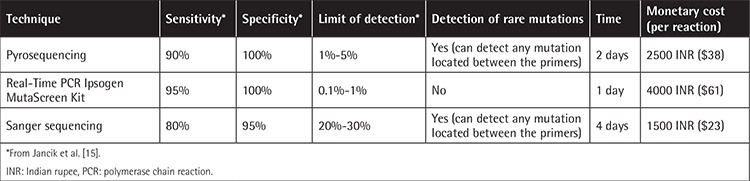
Sensitivity, specificity, time, and monetary cost of pyrosequencing, real-time polymerase chain reaction, and Sanger sequencing.
